# A 66-Year-Old Female with Apical Hypertrophic Cardiomyopathy Presenting with Hypertensive Crises and Type 2 Myocardial Infarction and a Normal Coronary Angiogram

**DOI:** 10.1155/2018/7089149

**Published:** 2018-10-25

**Authors:** Vineet Meghrajani, Karan Wats, Abhinav Saxena, Bilal Malik

**Affiliations:** ^1^Department of Internal Medicine, Maimonides Medical Center, 4802 10th Avenue, Brooklyn, New York 11219, USA; ^2^Department of Cardiology, Maimonides Medical Center, 4802 10th Avenue, Brooklyn, New York 11219, USA

## Abstract

A 66-year-old female presented to the emergency room with an episode of chest pain that lasted for a few minutes before resolving spontaneously. Electrocardiogram showed a left bundle branch block, left ventricular hypertrophy, and T wave inversions in the lateral leads. Initial cardiac troponin level was 0.15 ng/ml, with levels of 4 ng/ml and 9 ng/ml obtained 6 and 12 hours later, respectively. The peak blood pressure recorded was 195/43 mmHg. Echocardiogram with DEFINITY showed a small left ventricular cavity with apical hypertrophy, and coronary angiogram showed no stenotic or occluding lesions in the coronary arteries. The patient was admitted for a type 2 myocardial infarction with hypertensive crises. She was diagnosed with having apical hypertrophic cardiomyopathy, which is a variant of hypertrophic cardiomyopathy (HCM) in which the hypertrophy predominantly involves the apex of the left ventricle resulting in midventricular obstruction, as opposed to the left ventricular outflow tract obstruction seen in HCM. Patients with apical HCM may present with angina, heart failure, myocardial infarction, syncope, or arrhythmias and are typically managed with medications like verapamil and beta-blockers for those who have symptoms and antiarrhythmic agents like amiodarone and procainamide for treatment of atrial fibrillation and ventricular arrhythmias. An implantable cardioverter defibrillator (ICD) is recommended for high-risk HCM patients with a history of previous cardiac arrest or sustained episodes of ventricular tachycardia, syncope, and a family history of sudden death.

## 1. Introduction

There are several causes of troponin elevation other than acute coronary syndromes. Some of the common noncoronary ischemia-related causes include chronic renal failure, advanced heart failure, acute pulmonary embolism, acute inflammatory myocarditis, and coronary artery vasospasm. Apical hypertrophic cardiomyopathy (HCM) is one of the rare cardiomyopathies which can also present like an acute myocardial infarction (MI). We present a case of 66-year-old female who presented with chest pain to our emergency room and was found to have apical hypertrophic cardiomyopathy.

## 2. Case Presentation

**Figure 1:**
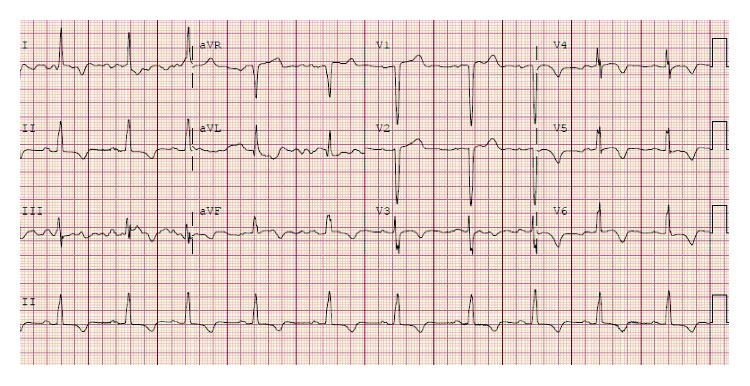
Initial EKG showing normal sinus cardiac rhythm with a left bundle branch block, possible left ventricular hypertrophy, and T wave inversions in the lateral leads. No ST segment changes were noted.

**Figure 2:**
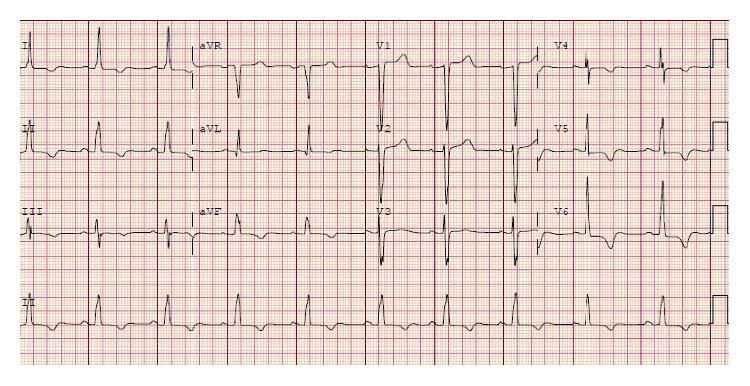
Follow-up EKG showing no changes compared to the first EKG.

**Figure 3:**
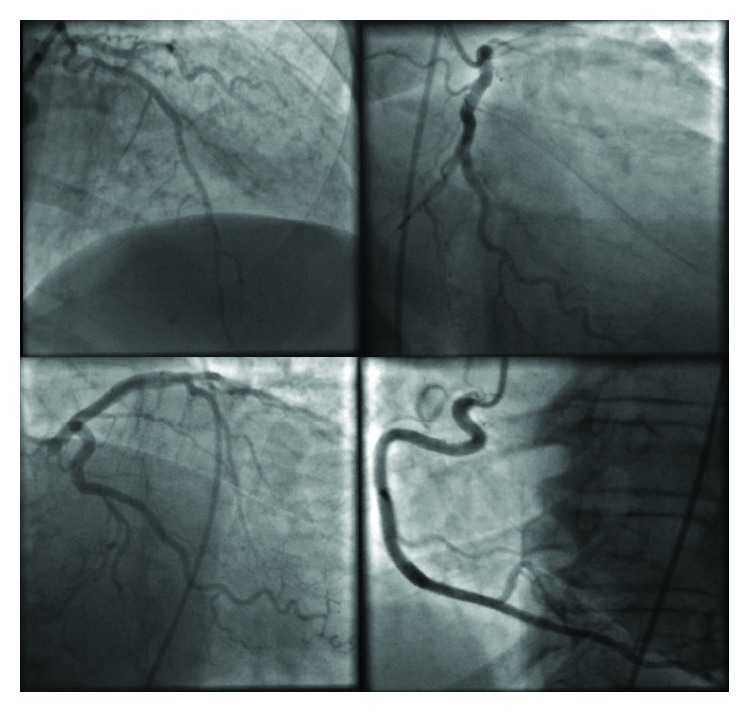
Coronary angiogram showing normal left main, left anterior descending, left circumflex, and right coronary arteries with no stenotic or occluding lesions.

**Figure 4:**
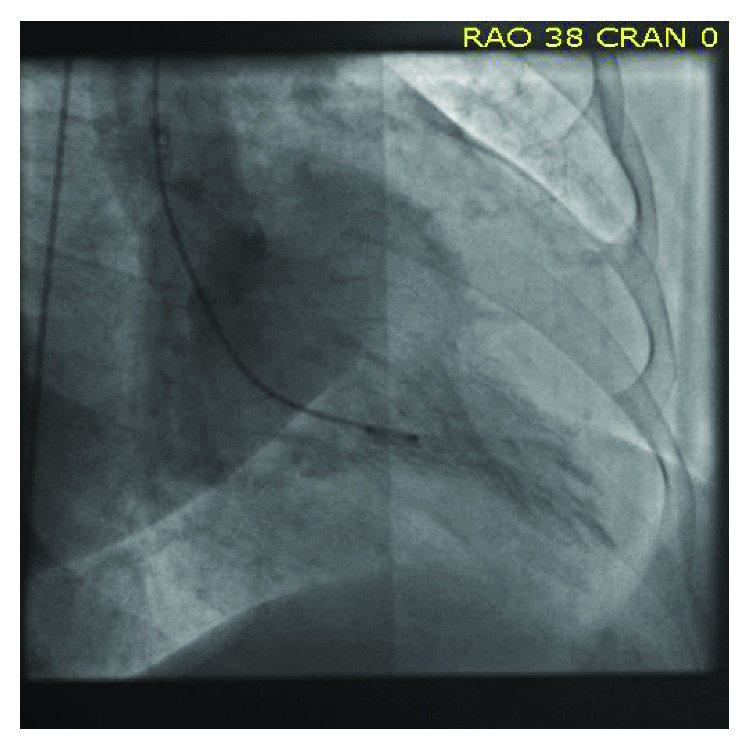
Cardiac left ventriculogram done by cardiac chamber catheterization confirming the echocardiographic finding of apical hypertrophy.

A 66-year-old independently functioning woman presented to the emergency room with an episode of midepigastric and left sternal chest pain. Her medical history included hypertension, hyperlipidemia, glaucoma, and multiple previous episodes of chest pain similar to her current episode that necessitated three separate coronary angiograms which showed no stenotic or occluding lesions in the coronary arteries. The patient described her chest pain as a sensation of burning that started suddenly at 11 pm in the night while she was resting comfortably at home after having dinner. The pain was mild in intensity, nonradiating, and lasted for a few minutes before resolving spontaneously. She denied having any dyspnea, palpitations, dizziness, or loss of consciousness during this episode. She had no history of smoking or illicit drug use. Her home medications included felodipine extended release 5 mg once daily, isosorbide mononitrate extended release 30 mg once daily, atorvastatin 80 mg once daily, losartan 100 mg once daily, hydrochlorothiazide 25 mg once daily, and metoprolol succinate extended release 100 mg once daily. The patient activated emergency medical services immediately after onset of her symptoms, who brought the patient to the emergency room. On arrival in the emergency room, the patient was asymptomatic. Her vital signs were as follows: blood pressure of 168/46 mmHg (right arm, supine position), heart rate of 66/min, respiratory rate of 19/min, and an oral temperature of 97.9 F. An electrocardiogram was obtained which showed a normal sinus cardiac rhythm with a left bundle branch block, possible left ventricular hypertrophy, and T wave inversions in the lateral leads. No ST segment changes were noted (Figure 1). There were no prior electrocardiograms available for comparison. Her laboratory data included a cardiac troponin level of 0.15 ng/ml. Follow-up cardiac troponin levels obtained 6 and 12 hours later were 4 ng/ml and 9 ng/ml, respectively. The patient continued to be asymptomatic during this time, and a follow-up EKG showed no changes compared to the first EKG (Figure 2). Her blood pressure, however, continued to remain high during her time in the emergency room with a peak blood pressure recording of 195/43 mmHg. Based on the elevated troponin level up to 9 ng/ml with the absence of ST segment changes on the EKG and the significantly elevated blood pressure, it was decided to admit the patient to the cardiac intensive care unit for treatment of a hypertensive crisis with elevated troponin levels possibly due to a type 2 myocardial infarction. She was given a loading dose of aspirin 325 mg and clopidogrel 300 mg orally and was started on an intravenous heparin drip at 12 units/kg/hour. A onetime dose of hydralazine 5 mg was administered intravenously which was followed by an improvement in the blood pressure to 150/46 mmHg. An echocardiogram was ordered with DEFINITY which showed a small left ventricular cavity with apical hypertrophy, an impaired relaxation pattern during left ventricular diastolic filling, and a left ventricular ejection fraction of 76–80%. The patient subsequently underwent a coronary angiogram the next day through right femoral artery access which showed normal left main, left anterior descending, left circumflex, and right coronary arteries with no stenotic or occluding lesions (Figure 3). A cardiac left ventriculogram was done by cardiac chamber catheterization during the procedure, which confirmed the echocardiographic finding of apical hypertrophy (Figure 4). Based on the finding of apical hypertrophic cardiomyopathy, it was decided to start the patient on metoprolol succinate extended release 100 mg daily and verapamil sustained release 180 mg daily. The remainder of the patient's hospitalization was uneventful, with an optimally controlled blood pressure and a downtrend in her cardiac troponin levels, and she was subsequently discharged on the 3rd hospital day.

## 3. Case Discussion

Apical hypertrophic cardiomyopathy is a variant of hypertrophic cardiomyopathy in which the hypertrophy of the myocardium predominantly involves the apex of the left ventricle resulting in midventricular obstruction, as opposed to the left ventricular outflow tract obstruction seen in HCM [1–3]. It occurs due to autosomal dominant mutations in one of nine genes encoding sarcomeric proteins, most commonly the sarcomere gene ACTC 1 (actin, alpha, cardiac muscle 1)—Glu101Lys missense mutation [4, 5]. Apical HCM is also known as Yamaguchi syndrome after the person who first described its imaging features in Japan and is more common in East Asian populations—representing 41 percent of Chinese patients with HCM and 15 percent of Japanese patients, but only 1–3 percent of non-Asian populations with HCM [1, 6–8]. The mean age at presentation has been variable in different studies, it was 41.4 ± 14.5 years in one study [3] and 58 ± 17 years in another [9]. Most patients with apical HCM are asymptomatic, and those who do develop symptoms usually present with angina, heart failure, myocardial infarction, presyncope, syncope, atrial fibrillation, or ventricular fibrillation. These occur usually due to diastolic dysfunction with low cardiac output [3]. The typical clinical and diagnostic features of apical HCM include audible fourth heart sound, “giant” negative T waves on EKG, particularly in left precordial leads, apical wall motion abnormalities including hypokinesis and aneurysm formation, and “spade-like” configuration of left ventricular cavity at end-diastole on imaging [3, 10, 11]. Among these findings, the “giant” negative T wave has been identified as a predictor of favorable outcome [8]. The diagnosis of apical HCM can be made with echocardiography, left ventriculography, computed tomography, or most accurately with cardiac MRI [1, 12, 13]. Lladó et al. showed that MRI allowed better overall assessment of the degree and extent of LVH in HCM patients than did echocardiogram [12]. The apical segment was adequately visualized in all patients by MRI but only in 60% of the patients by echo, and MRI allows the early diagnosis of apical HCM by identifying even a small quantity of hypertrophied myocardium in different apical locations in a “nonspade” variety of apical HCM [12, 14]. While apical HCM has a better mortality prognosis than other forms of HCM, with one retrospective study of 105 patients having a total cardiovascular mortality rate of 1.9% over 13.6 ± 8.3 years [3] and overall estimated survival of 95 percent at 15 years, it is still associated with a relatively high rate of important cardiac events—30 percent of patients experienced a serious cardiac complication, most commonly atrial fibrillation (12 percent) or a myocardial infarction (10 percent) [3]. In the same study, apical myocardial infarction in the presence of normal coronary arteries, a presentation similar to the patient in our case, was found in about 10% of patients. The possible mechanisms responsible for ischemia in apical HCM include limitation in coronary flow reserve, decreased capillary myocardial ratio, small vessel disease, and other mechanisms [15–17]. Severe clinical manifestations, including sudden cardiac death, severe arrhythmias, and apical infarction with apical aneurysm, are uncommon with apical HCM, although they have been described [3]. Among the subtypes of HCM, the “true apical” phenotype (hypertrophy of only the apical segments) has been shown to have improved survival over a “distal-dominant” phenotype (cases in which hypertrophy extended into the middle LV segments) [18]. In another study of 193 patients in Minnesota, the observed versus expected 20-year survival was 47% versus 60% [9]. Sudden cardiac death, resuscitated cardiac arrest, and/or defibrillator discharge was observed in 6% of patients during follow-up.

While apical HCM is low on the list of suspected differentials for patients presenting with chest pain and elevated cardiac troponin levels on laboratory testing, the more common causes of such a presentation being STEMI or NSTEMI, this case still demonstrates the importance of following the proper testing protocol for patients who present with these findings to the emergency room—namely, an EKG, followed by testing for cardiac troponin levels, a bedside echocardiogram, and an urgent coronary angiogram if EKG changes are seen along with elevated cardiac troponin levels. For patients with no EKG changes and elevation in cardiac troponin levels, they should still be treated as an NSTEMI with anticoagulation and an early coronary angiogram preferably within 24 hours of presentation, especially if they have clinical findings that are strongly suggestive of an NSTEMI, i.e., symptoms of ischemic chest pain on presentation and prior risk factors of coronary artery disease.

The approach to the management of apical HCM is the same as for most patients with HCM; symptomatic patients are initially treated with medical therapy, which includes medications like verapamil and beta-blockers [3, 19, 20]. Antiarrhythmic agents such as amiodarone and procainamide are used in the treatment of atrial fibrillation and ventricular arrhythmias [21]. Since apical HCM does not involve LVOT obstruction, septal reduction therapy is almost never used to treat apical HCM. Similarly, the risk of ventricular tachyarrhythmias and sudden cardiac death is low in apical HCM, and an implantable cardioverter defibrillator (ICD) is only recommended for high risk HCM patients with a history of previous cardiac arrest or sustained episodes of ventricular tachycardia, syncope, a family history of sudden death, or episodes of nonsustained ventricular tachycardia on serial Holter monitoring [22].

## 4. Conclusion

Apical HCM is a variant of HCM in which the hypertrophy of the myocardium predominantly involves the apex of the left ventricle resulting in midventricular obstruction, as opposed to the left ventricular outflow tract obstruction seen in HCM. While apical HCM has a better mortality prognosis than other forms of HCM, it is still associated with a relatively high rate of important cardiac events like atrial fibrillation or myocardial infarction. Apical HCM is low on the list of suspected differentials for patients presenting with chest pain and elevated cardiac troponin levels on laboratory testing, the more common causes of such a presentation being STEMI or NSTEMI. This case demonstrates the importance of following the proper testing protocol for patients who present with these findings to the emergency room—namely, an EKG, followed by testing for cardiac troponin levels, a bedside echocardiogram, and an urgent coronary angiogram if EKG changes are seen along with elevated cardiac troponin levels. The approach to the management of apical HCM is similar to most patients with HCM—medical therapy for symptomatic patients, which includes medications like verapamil and beta-blockers and antiarrhythmic agents such as amiodarone and procainamide for treatment of atrial fibrillation and ventricular arrhythmias. Since apical HCM does not involve LVOT obstruction, septal reduction therapy is almost never used to treat apical HCM. The risk of ventricular tachyarrhythmias and sudden cardiac death is low in apical HCM, and an implantable cardioverter defibrillator (ICD) is only recommended for high-risk HCM patients with a history of previous cardiac arrest or sustained episodes of ventricular tachycardia, syncope, a family history of sudden death, or episodes of nonsustained ventricular tachycardia on serial Holter monitoring.
